# Stochastic Gene Expression Revisited

**DOI:** 10.3390/genes12050648

**Published:** 2021-04-26

**Authors:** Andrzej Tomski, Maciej Zakarczemny

**Affiliations:** 1 Institute of Mathematics, University of Silesia in Katowice, 40-007 Katowice, Poland; andrzej.tomski@us.edu.pl; 2 Department of Applied Mathematics, Faculty of Computer Science and Telecommunications, Cracow University of Technology, 31-155 Cracow, Poland

**Keywords:** stochastic gene expression, pre-mRNA, iterated function system, limit measure, gene expression process simulation, 47D06

## Abstract

We investigate the model of gene expression in the form of Iterated Function System (IFS), where the probability of choice of any iterated map depends on the state of the phase space. Random jump times of the process mark activation periods of the gene when pre-mRNA molecules are produced before mRNA and protein processing phases occur. The main idea is inspired by the continuous-time piecewise deterministic Markov process describing stochastic gene expression. We show that for our system there exists a unique invariant limit measure. We provide full probabilistic description of the process with a comparison of our results to those obtained for the model with continuous time.

## 1. Introduction

An interesting problem in the field of modeling of biological processes [1] has been to understand the interactions in gene regulatory networks. Information on various approaches to describe relations between genes can be found in the paper [2]. Numerous methods based on chemical networks [3], logical networks [4] or dynamical systems [5] are used. As [6] suggests, piecewise deterministic stochastic processes can be used to may model genetic patterns. Our paper belongs to this methodology, but it investigates a discrete-time analogue of the ordinary differential equation case. A more common approach would be to use Markov jump processes, which lead to chemical master equations (CME) considered in discrete state spaces [7]. There are several methods to solve CME’s, including finding exact solution (i.e., by means of Poisson representation) or approximation methods. Unfortunately, all these methods can only approximate the solution of the CME or they can be applied in particular cases. Moreover, most of related studies generally focus on the translation phase, without putting any importance to the transcription phase or the intermediate mRNA processing. The main advantage of the analysis derived from piecewise deterministic stochastic processes is the potential to extend a model simply by adding new types of particles to the stochastic reaction network. Our approach, dependent on piecewise deterministic stochastic process combines deterministic approach represented by dynamical systems with stochastic effects represented by Markov processes. In many cases, discrete time or continuous-time dynamical systems became two alternative ways to describe the dynamics of a network. The formalism of discrete-time systems does not concentrate on instantaneous changes in the level of gene expression but rather on the overall change in a given time interval. This may be the right approach to model processes where some reactions must be integrated over a short timeline for the purpose of revealing more important interactions affecting expression levels with respect to a larger time perspective. Another aspect is that the experimental data obtained from living cells are undoubtedly discrete in time and because of the costs we are limited only to relatively small sets of samples [8]. In recent years, difference equation models appeared (see [9,10,11]). In this work we concentrate on the gene expression process with four stages: activation of the gene, being followed then by pre-mRNA and mRNA and protein processing [12].

Basically, after a gene is activated at a random time moment, mature mRNA is produced in the nucleus, then it is transported to the cytoplasm, where the protein translation follows. However, it is known that translated mRNA molecules must get through further processing first, before a new protein particle is formed. Besides that, many sources [13] claim that at least one additional phase, primary transcript (pre-mRNA) processing also takes place. Actually, in the world of eukaryotic genes, after activation at a random time point, the DNA is transformed into some certain pre-mRNA form of transcript. Then, the non-coding sequences (introns) of transcript are removed and the coding (exons) regions are combined. This process is called mRNA splicing. In addition to the splicing step, pre-mRNA processing also includes at least three other processes: addition of the m7G cap at the $5^{\prime }$ end to increase the stability, polyadenylation at the $3^{\prime }$-UTR which affects the miRNA regulation and RNA degradation, and post-transcriptional modifications (methylation). In some genes, there is an extra step of RNA editing. Multiple other genetic modifications take place under the general term called RNA processing. In such a situation we finally get a functional form of mRNA, which is transferred into the cytoplasm, where in the translation phase, mRNA is decoded into a protein. Of course, both mRNA and protein undergo biological degradation. The presence of a random component in our model, responsible for switching between active and inactive states of the gene in the random time moments has been identified in the continuous case as a piecewise deterministic Markov process (PDMP) [14]. This class of stochastic processes can be considered to be randomly switching dynamical systems with the intensities of the consecutive jumps dependent on the current state of the phase. However, if we consider discrete-time scale, then we must investigate iterated function systems (IFS’s) with place-dependent probabilities, see [15] or [16]. We are going to unify a common approach for both time continuous and time discrete dynamical systems with random jumps. We will investigate the existence of stationary distributions for time discrete dynamical systems with random jumps and compare its form with the continuous-time case. Here we introduce jump intensity functions, which play crucial role in the distribution of waiting time for the jump [17] and for this purpose we provide an appropriate cumulative distribution function. Specifically, instead of $\exp\{-\int_{0}^{t}q\left(\pi \left(s,x_{0}\right)\right)\;ds\}$ in the continuous case (see [17]), we justify the formula for the life-span function $\exp\{-\sum_{s=0}^{\lfloor t\rfloor -1}q\left(\pi \left(s,x_{0}\right)\right)\}$ in the discrete case. In this way we obtain certain IFS corresponding to a discrete-time Markov process with jumps characterized by jump intensity functions.

A consequence of the stochastic expression is the diversity of the population in terms of the composition of individual proteins and gene expression profiles [13]. Stochastic gene expression causes expression variability within the same organism or tissue, which has effect on biological function.

This work is organized as follows. In Section 3 we present the model, and we give the definition of our process. In Section 4 and Section 5 we investigate its properties and we describe it as an IFS with place-dependent probabilities. In Section 6, we use the classical result of Barnsley [18], to show that our process converges in distribution to a unique invariant measure when the number of iterations converges to infinity and we describe the properties of this measure in Section 7. A complete step-by-step description of the whole process, summing up all the information from the earlier sections, is provided in Section 8. A computer simulation of trajectories of the process, is the content of Section 9, with the source code available in GitHub [19]. In Section 10 presents the derivation of formulas for the support of the invariant measure. Summary is the last section of this paper.

## 2. Methods

In this paper, we investigate a model which is based on IFS with place-dependent probabilities. Compared to the model presented in [17], we replace ordinary differential equations (ODEs) by the system of difference equations, which leads to the investigation of discrete-time model, but can be generalized into continuous case. The question therefore arises how we justify the usage of difference equations in our model. Our justification is that our results remain consistent with the work from [17] which can be considered as an alternative way of description of such systems. Discrete approach is an attempt to mathematical formulation of the problem using different tools. This paper provides discussion between different approaches (sometimes different than standard accepted principles).

## 3. Stochastic Gene Expression—Discrete Case

Gene expression is a very complex biological process including multiple essential subprocesses. In the continuous case, Lipniacki et al. [6] introduced a model based mathematically on piecewise deterministic Markov process which includes three crucial phases: gene activation, mRNA and protein processing.

Let $\xi_{1}\left(t\right)$ denote the number of pre-mRNA molecules at time t, $\xi_{2}\left(t\right)$ denote the number of mRNA molecules at time t, $\xi_{3}\left(t\right)$ denote the number of protein molecules at time t, where in general t∈[0,∞). Analogically to the solution of continuous model, we can introduce the symbol $\pi_{\Delta t}^{i}\left(\xi \left(t\right)\right)$, where $\xi \left(t\right)=(\xi_{1}\left(t\right),\xi_{2}\left(t\right),\xi_{3}\left(t\right))$. A discrete-time model would evaluate $\pi_{\Delta t}^{i}\left(\xi \left(t\right)\right)$ after Δt starting from ξ(t).

The difference equation then could be given by equation of the following kind:

$$\Delta \xi \left(t\right)=\pi_{\Delta t}^{i}\left(\xi \left(t\right)\right)-\xi \left(t\right)=\xi \left(t+\Delta t\right)-\xi \left(t\right).$$

**Figure 1:**
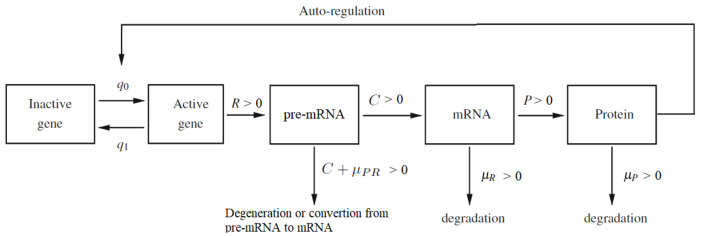
The diagram of auto-regulated gene expression with pre-mRNA, mRNA and protein contribution. Description of the parameters: $q_{0}$ and $q_{1}$ are switching intensity functions; *R* is the speed of synthesis of pre-mRNA molecules if the gene is active; *C* is the rate of converting pre-mRNA into active mRNA molecules; *P* is the rate of converting mRNA into protein molecules; $\mu_{PR}$ is the pre-mRNA degradation rate; $\mu_{R}$ is the mRNA degradation rate; $\mu_{P}$ is the protein degradation rate. The sum $C+\mu_{PR}$ should be treated as a total degradation rate of the pre-mRNA particles.

Thus, our approach is based on particular translation Δt. In the paper we fix the value of Δt. For the sake of simplicity, we denote Δt=δ. Let $f:R\to R^{3}$, typically in the theory of linear difference equations, we define Δf(t)=f(t+1)−f(t). In our model, we take Δf(t)=f(t+δ)−f(t), hence δ is a time step, instead of unity. Please note that one could use the basic techniques of scaling variables to get unity, instead of δ. We consider the following model being represented by the system of difference equations in the form.

(1) $$\left\{\begin{array}{c} \Delta \xi_{1}\left(t\right)=\xi_{1}\left(t+\delta \right)-\xi_{1}\left(t\right)=R\gamma \left(t\right)-\left(C+\mu_{PR}\right)\xi_{1}\left(t\right)\\ \Delta \xi_{2}\left(t\right)=\xi_{2}\left(t+\delta \right)-\xi_{2}\left(t\right)=C\xi_{1}\left(t\right)-\mu_{R}\xi_{2}\left(t\right)\\ \Delta \xi_{3}\left(t\right)=\xi_{3}\left(t+\delta \right)-\xi_{3}\left(t\right)=P\xi_{2}\left(t\right)-\mu_{P}\xi_{3}\left(t\right), \end{array}\right.$$

where t≥0,t∈δZ; R>0 is the speed of synthesis of pre-mRNA molecules if the gene is active; C>0 is the rate of converting pre-mRNA into active mRNA molecules; $\mu_{PR}>0$ is the pre-mRNA degradation rate; $\mu_{R}>0$ is the mRNA degradation rate; P>0 is the rate of converting mRNA into protein molecules and $\mu_{P}>0$ is the protein degradation rate (see Figure 1). Provided the time step δ is small enough, $\mu_{PR}$, $\mu_{R}$, $\mu_{P}$, *R*, *C*, and *P* will be independent of δ, see [10]. Here γ(t):[0,∞)→{0,1} such that

(2) $$\gamma \left(t\right)=\left\{\begin{array}{cc} i & \mathrm{if}\;t\in [0,t_{1})\\ 1-i & \mathrm{if}\;t=t_{1} \end{array}\right.,$$

where $t_{1}$ denotes the moment of first jump of this process, where the distribution of $t_{1}$ is described by life-span function in Section 3.1.

The values of the coefficients are scaled simultaneously to the interval [0,1] so that their relative importance in the model can be more easily seen. Basically, there is no biological reason behind imposing any restrictions on the values of the parameters. However, we need this step to perform mathematical analysis of the asymptotic behavior of this system. We can transform almost any system in such way. An exception is the case when the system (1) reduces to less than three equations. It can happen, when some coefficients are equal to zero or they are equal one to another. We do not analyze such cases here. An open question then remains, what happens, for example, when $C+\mu_{PR}=\mu_{R}$ or $\mu_{R}+P=1$ and other similar situations, described below. To avoid degenerate cases, we will assume in the model (1) that:

(3) $$0<C+\mu_{PR}<1,\;0<\mu_{R}+P<1,\;0<\mu_{P}<1,$$

since the number of degraded molecules cannot exceed the current number of corresponding molecules.

If we assume that γ(t)≡i∈{0,1}=I, we obtain the following system of linear difference equations:

(4) $$\left\{\begin{array}{c} \Delta \xi_{1}\left(t\right)=Ri-\left(C+\mu_{PR}\right)\xi_{1}\left(t\right)\\ \Delta \xi_{2}\left(t\right)=C\xi_{1}\left(t\right)-\mu_{R}\xi_{2}\left(t\right)\\ \Delta \xi_{3}\left(t\right)=P\xi_{2}\left(t\right)-\mu_{P}\xi_{3}\left(t\right), \end{array}\right.$$

with initial condition $\bar{\xi }=\left(\xi_{1}\left(0\right),\xi_{2}\left(0\right),\xi_{3}\left(0\right)\right),$ where t∈δZ,t≥0.

Please note that $\Delta \xi_{k}\left(t\right)=\xi_{k}\left(t+\delta \right)-\xi_{k}\left(t\right),k\in \left\{1,2,3\right\}$.

**Example** **1.** *Let us consider the system (4) with the values of parameters $R=1,\mu_{PR}=\frac{1}{4}$, $C=\mu_{R}=\frac{1}{4},P=\mu_{P}=\frac{1}{3}.$ In this case, the solutions of (4) are:*

(5) $$\left\{\begin{array}{c} \xi_{1}\left(t\right)={\left(\frac{1}{2}\right)}^{\frac{t}{\delta }}\left(\xi_{1}\left(0\right)-2i\right)+2i\\ \xi_{2}\left(t\right)={\left(\frac{3}{4}\right)}^{\frac{t}{\delta }}\left(\xi_{2}\left(0\right)+\xi_{1}\left(0\right)-4i\right)-{\left(\frac{1}{2}\right)}^{\frac{t}{\delta }}\left(\xi_{1}\left(0\right)-2i\right)+2i\\ \xi_{3}\left(t\right)={\left(\frac{2}{3}\right)}^{\frac{t}{\delta }}\left(\xi_{3}\left(0\right)-4\xi_{2}\left(0\right)-6\xi_{1}\left(0\right)+18i\right)+\\ +{\left(\frac{3}{4}\right)}^{\frac{t}{\delta }}\left(4\xi_{2}\left(0\right)+4\xi_{1}\left(0\right)-16i\right)+{\left(\frac{1}{2}\right)}^{\frac{t}{\delta }}\left(2\xi_{1}\left(0\right)-4i\right)+2i. \end{array}\right.$$

*Please note that this formula is valid for any t∈R, hence we could also extend the solutions (5) to the continuous-time case.*

**Figure 2:**
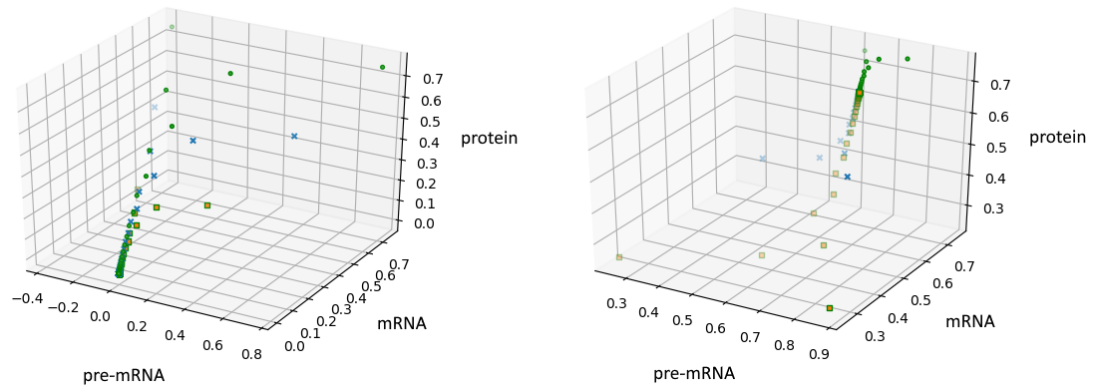
A solution of Equation (4) for $\delta =1,R=1,\mu_{PR}=\frac{1}{4},C=\mu_{R}=\frac{1}{4},P=\mu_{P}=\frac{1}{3}$ with i=0 on the left and i=1 on the right.

In the Figure 2, we show these trajectories for i=0 (left panel) and i=1 (right panel).

If we assume that i∈I is constant, then the system (1) takes the form (4) which can be rewritten in the following form:

(6) $$\left\{\begin{array}{c} \xi_{1}\left(t+\delta \right)=Ri-\left(C+\mu_{PR}-1\right)\xi_{1}\left(t\right)\\ \xi_{2}\left(t+\delta \right)=C\xi_{1}\left(t\right)-\left(\mu_{R}-1\right)\xi_{2}\left(t\right)\\ \xi_{3}\left(t+\delta \right)=P\xi_{2}\left(t\right)-\left(\mu_{P}-1\right)\xi_{3}\left(t\right), \end{array}\right.$$

with the initial condition $\bar{\xi }=\left(\xi_{1}\left(0\right),\xi_{2}\left(0\right),\xi_{3}\left(0\right)\right).$

**Remark** **1.** *We assumed that i is constant, but important is to explain the way our process behaves after the next switch.*

*For the purpose of calculation of $\left(\xi_{1}\left(t+\delta \right),\;\xi_{2}\left(t+\delta \right),\;\xi_{3}\left(t+\delta \right)\right)$ we need to use the value of i(t), not i(t+δ), in consistency with the formula (1).*

If (3) hold and $C+\mu_{PR}\neq \mu_{R},\;\mu_{R}\neq \mu_{P},\;\mu_{P}\neq C+\mu_{PR}$, then the solutions of the system (4) are:

(7) $$\left\{\begin{array}{c} \xi_{1}\left(t\right)={\left(1-C-\mu_{PR}\right)}^{\frac{t}{\delta }}\left(\xi_{1}\left(0\right)-\frac{R}{C+\mu_{PR}}i\right)+\frac{R}{C+\mu_{PR}}i,\\ \xi_{2}\left(t\right)={\left(1-\mu_{R}\right)}^{\frac{t}{\delta }}\left(\xi_{2}\left(0\right)+\frac{C}{C+\mu_{PR}-\mu_{R}}\xi_{1}\left(0\right)-\frac{RC}{\left(C+\mu_{PR}-\mu_{R}\right)\mu_{R}}i\right)+\\ \;\;\;+\;{\left(1-C-\mu_{PR}\right)}^{\frac{t}{\delta }}\left(-\frac{C}{C+\mu_{PR}-\mu_{R}}\xi_{1}\left(0\right)+\frac{RC}{\left(C+\mu_{PR}-\mu_{R}\right)\left(C+\mu_{PR}\right)}i\right)+\frac{RC}{\left(C+\mu_{PR}\right)\mu_{R}}i,\\ \xi_{3}\left(t\right)={\left(1-\mu_{P}\right)}^{\frac{t}{\delta }}\times \\ \;\;\;\times \left(\xi_{3}\left(0\right)+\frac{P}{\mu_{R}-\mu_{P}}\xi_{2}\left(0\right)+\frac{PC}{\left(\mu_{R}-\mu_{P}\right)\left(C+\mu_{PR}-\mu_{P}\right)}\xi_{1}\left(0\right)-\frac{PCR}{\left(\mu_{R}-\mu_{P}\right)\left(C+\mu_{PR}-\mu_{P}\right)\mu_{P}}i\right)+\\ \;\;\;+\;{\left(1-\mu_{R}\right)}^{\frac{t}{\delta }}\times \\ \;\;\;\times \left(-\frac{P}{\mu_{R}-\mu_{P}}\xi_{2}\left(0\right)-\frac{PC}{\left(\mu_{R}-\mu_{P}\right)\left(C+\mu_{PR}-\mu_{R}\right)}\xi_{1}\left(0\right)+\frac{PCR}{\left(C+\mu_{PR}-\mu_{R}\right)\left(\mu_{R}-\mu_{P}\right)\mu_{R}}i\right)+\\ \;\;\;+\;{\left(1-C-\mu_{PR}\right)}^{\frac{t}{\delta }}\times \\ \;\;\;\times \left(\frac{PC}{\left(C+\mu_{PR}-\mu_{R}\right)\left(C+\mu_{PR}-\mu_{P}\right)}\xi_{1}\left(0\right)-\frac{PCR}{\left(C+\mu_{PR}-\mu_{R}\right)\left(C+\mu_{PR}-\mu_{P}\right)\left(C+\mu_{PR}\right)}i\right)+\\ \;\;\;+\frac{PCR}{\left(C+\mu_{PR}\right)\mu_{R}\mu_{P}}i. \end{array}\right.$$

We can extend this formula from t∈δZ,t≥0 to t∈[0,∞), since the formula (7) is valid not only for t∈δZ,t≥0 but also for t∈[0,∞).

Using the formula (7) we denote by

(8) $$\pi^{i}\left(t,\bar{\xi }\right)=\left(\xi_{1}\left(t\right),\xi_{2}\left(t\right),\xi_{3}\left(t\right)\right)$$

the solutions of the system (6), where we assume that t∈[0,∞). Also note that:

(9) $$\pi^{0}\left(t,w-\bar{\xi }\right)=w-\pi^{1}\left(t,\bar{\xi }\right),$$

where $w=\left(\frac{R}{C+\mu_{PR}},\frac{RC}{\left(C+\mu_{PR}\right)\mu_{R}},\frac{PCR}{\left(C+\mu_{PR}\right)\mu_{R}\mu_{P}}\right)$. One can rewrite (9) in the following form:

(10) $$\pi^{1}\left(t,\bar{\xi }\right)=w-\pi^{0}\left(t,w\right)+\pi^{0}\left(t,\bar{\xi }\right),$$

see also [17]. For the rest of the paper we assume $C+\mu_{PR}\neq \mu_{R},\;\mu_{R}\neq \mu_{P}$ and $\mu_{P}\neq C+\mu_{PR}$ (to avoid “degenerative” cases).

After substituting $a=1-C-\mu_{PR},\;b=1-\mu_{R},\;c=1-\mu_{P},$ where a,b,c∈(0,1),a≠b,b≠c and c≠a in the system (6) and taking:

(11) $$\left\{\begin{array}{c} \xi_{1}\left(t\right)=R\left(a-c\right)\left(a-b\right)\xi_{1}^{*}\left(t\right)\\ \xi_{2}\left(t\right)=CR\left(a-c\right)\xi_{1}^{*}\left(t\right)+CR\left(b-c\right)\xi_{2}^{*}\left(t\right)\\ \xi_{3}\left(t\right)=PCR\left(\xi_{1}^{*}\left(t\right)+\xi_{2}^{*}\left(t\right)+\xi_{3}^{*}\left(t\right)\right), \end{array}\right.$$

we obtain equivalent system of difference equations:

(12) $$\left\{\begin{array}{c} \xi_{1}^{*}\left(t+\delta \right)=a\xi_{1}^{*}\left(t\right)+\frac{1}{\left(a-c)(a-b\right)}i\\ \xi_{2}^{*}\left(t+\delta \right)=b\xi_{2}^{*}\left(t\right)+\frac{1}{\left(b-a)(b-c\right)}i\\ \xi_{3}^{*}\left(t+\delta \right)=c\xi_{3}^{*}\left(t\right)+\frac{1}{\left(c-a)(c-b\right)}i, \end{array}\right.$$

with initial condition $\bar{\xi }=\left(\xi_{1}\left(0\right),\xi_{2}\left(0\right),\xi_{3}\left(0\right)\right)\in R^{3}$. We will return to system (12) in Section 4 and Section 10.

### 3.1. Life-Span Function

Let *f* be a function defined on the set of non-negative integers $Z_{\geq 0}$ with values in $R^{d},\;d\in N$. We define Δf(k)=f(k+1)−f(k). Analogically to description from [20] we investigate the following system of equations:

(13) $$\Delta x\left(t\right)=g\left(x\left(t\right)\right),\;\mathrm{where}\;\;x:Z_{\geq 0}\to R^{d}\;\;\mathrm{and}\;\;g:R^{d}\to R^{d}.$$

Let $t_{0}=0$ and $t_{n}$ be a time when the process changes its state n−th time, $t_{n}\in Z_{\geq 0}$, $t_{n+1}>t_{n}$. Let $x\left(t\right),t\in \left[t_{n-1},\;t_{n}\right)\cap Z_{\geq 0}=\left\{t_{n-1},t_{n-1}+1,\ldots ,t_{n}-1\right\}$ be a discrete trajectory of the process from time $t_{n-1}$ to $t_{n}$. Let $\pi (t,x_{0})$ be a solution of the Equation (13) with the initial condition $x\left(0\right)=x_{0}$. Now we define q(x) as the intensity function with parameter *x* which means that after small fixed natural time Δt our process changes its state with probability q(x)Δt. Let *B* be a Borel subset of $R^{3}$ and

(14) $$P\left(x,B\right)=Prob(x\left(t_{n}\right)\in B|x\left(t_{n}-1\right)=x).$$

For any n>0 the distribution function of the difference $t_{n}-t_{n-1}$ is given by $F\left(t\right)=1-\Phi_{x_{0}}\left(t\right)$, where $\Phi_{x_{0}}\left(t\right)=Prob\left(t_{n}-t_{n-1}>t\right)$ is a survival function, i.e., the probability of duration between consecutive changes of states by the process.

Please note that $\Phi_{x_{0}}\left(0\right)=1,\;F\left(0\right)=0$. If n=1, then $\Phi_{x_{0}}\left(t\right)$ is a probability that the process will change its state for the first time after time t. Then we have

(15) $$\begin{array}{c} Prob\left(t\leq t_{1}\leq t+\Delta t|t_{1}>t\right)=\frac{\Phi_{x_{0}}\left(t\right)-\Phi_{x_{0}}\left(t+\Delta t\right)}{\Phi_{x_{0}}\left(t\right)}=q\left(\pi \left(t,x_{0}\right)\right)\Delta t. \end{array}$$

Hence, by taking Δt=1 we obtain the following formulas:

(16) $$\frac{\Phi_{x_{0}}\left(t+1\right)}{\Phi_{x_{0}}\left(t\right)}=1-q\left(\pi \left(t,x_{0}\right)\right),\;\;\mathrm{and}\;\;\Phi_{x_{0}}\left(t\right)=\prod_{s=0}^{t-1}\left(1-q\left(\pi \left(s,x_{0}\right)\right)\right).$$

Therefore,

(17) $$\Phi_{x_{0}}\left(t\right)=\exp\left\{\sum_{s=0}^{t-1}\log\left(1-q\left(\pi \left(s,x_{0}\right)\right)\right)\right\}\approx \exp\left\{-\sum_{s=0}^{t-1}q\left(\pi \left(s,x_{0}\right)\right)\right\},$$

assuming $q(\pi \left(s,x_{0}\right))$ lies in the sufficiently small neighborhood of zero, since $\lim_{h\to 0}\frac{1}{h}\left(\log\left(1+h\right)\right)=1$. Similar formula has been derived in the continuous case (see 1.7 [20]), but in the Formula (17) we use sum instead of integral operator. Above considerations are justified by using the following definition.

**Definition** **1.** *We define life-span function by the following formula:*

(18) $$\Phi_{x_{0}}\left(t\right)=\exp\left\{-\sum_{s=0}^{t-1}q\left(\pi \left(s,x_{0}\right)\right)\right\},$$

*where $q:R^{d}\to R_{\geq 0}$ is a bounded switching intensity function and t is a non-negative integer number. In our case, if t∈R we can take*

(19) $$\Phi_{x_{0}}\left(t\right)=\exp\left\{-\sum_{s=0}^{\lfloor t\rfloor -1}q\left(\pi \left(s,x_{0}\right)\right)\right\}.$$

Hence, instead of $\exp\{-\int_{0}^{t}q\left(\pi \left(s,x_{0}\right)\right)\;ds\}$ in the continuous case (the formula used in the paper [17]), we justify the formula for the life-span function $\exp\{-\sum_{s=0}^{\lfloor t\rfloor -1}q\left(\pi \left(s,x_{0}\right)\right)\}$ in the discrete case.

### 3.2. Piecewise Deterministic Markov Process

In this subsection we introduce basic characteristics of the Markov process represented by the system (4) that will be needed for further considerations. Here, we assume that t∈[0,∞). Let $q_{0}\left(\xi_{1},\xi_{2},\xi_{3}\right)$ and $q_{1}\left(\xi_{1},\xi_{2},\xi_{3}\right)$ be positive and continuous functions on the set $R^{3}$. Let $\xi =\left(\xi_{1},\xi_{2},\xi_{3}\right)\in R^{3}$. Using our Definition 1 of the life-span function, we can define the distribution function of the difference $t_{n}-t_{n-1}$, namely

(20) $$F_{\xi ,i}\left(t\right)=1-\exp\left\{-\sum_{s=0}^{\lfloor t\rfloor -1}q_{i}\left(\pi^{i}\left(s,\xi \right)\right)\right\},$$

where as before, $t_{n}$ is a time when the process changes its state n−th time. Please note that $F_{\xi ,i}\left(0\right)=0$.

The explicit expressions for the solutions $\pi^{i}\left(t,\xi \right),\;t\in \left[0,\infty \right)$ of the system (4) were found in (7). Hence,

(21) $$\lim_{t\to \infty }\left(\pi^{i}\left(t,\xi \right)\right)=\left(\frac{R}{C+\mu_{PR}}i,\frac{RC}{\left(C+\mu_{PR}\right)\mu_{R}}i,\frac{PCR}{\left(C+\mu_{PR}\right)\mu_{R}\mu_{P}}i\right)=wi,$$

for the arbitrary choice of ξ. It is known [20] that such description gives us piecewise deterministic Markov process

(22) $$X_{t}=\left(\xi_{1}\left(t\right),\xi_{2}\left(t\right),\xi_{3}\left(t\right),i\left(t\right)\right)$$

on the state space $\left[0,\frac{R}{C+\mu_{PR}}\right]\times \left[0,\frac{RC}{\left(C+\mu_{PR}\right)\mu_{R}}\right]\times \left[0,\frac{PCR}{\left(C+\mu_{PR}\right)\mu_{R}\mu_{P}}\right]\times \left\{0,1\right\}$ with two switching intensity functions $q_{0}$, $q_{1}$ and the transition measure given by Dirac Delta Function concentrated at the point $\left(x_{1},x_{2},x_{3},1-i\right)$. Please note that by the definition of the system (1), the set $\left[0,\frac{R}{C+\mu_{PR}}\right]\times \left[0,\frac{RC}{\left(C+\mu_{PR}\right)\mu_{R}}\right]\times \left[0,\frac{PCR}{\left(C+\mu_{PR}\right)\mu_{R}\mu_{P}}\right]\times \left\{0,1\right\},$ is invariant with respect to the process $X_{t}$, i.e., if

$$X_{0}\in \left[0,\frac{R}{C+\mu_{PR}}\right]\times \left[0,\frac{RC}{\left(C+\mu_{PR}\right)\mu_{R}}\right]\times \left[0,\frac{PCR}{\left(C+\mu_{PR}\right)\mu_{R}\mu_{P}}\right]\times \left\{0,1\right\},$$

then

$$X_{t}\in \left[0,\frac{R}{C+\mu_{PR}}\right]\times \left[0,\frac{RC}{\left(C+\mu_{PR}\right)\mu_{R}}\right]\times \left[0,\frac{PCR}{\left(C+\mu_{PR}\right)\mu_{R}\mu_{P}}\right]\times \left\{0,1\right\}$$

for all t∈[0,∞).

The technical proof of this fact, which is based on the usage of formulas (7), is omitted. In the fourth chapter, we introduce Iterated Function Systems to investigate the existence of invariant measure and its support.

## 4. Iterated Function System

For i∈I we define the mappings $S_{i}:R^{3}\to R^{3}$ given by the formulas

(23) $$S_{i}\left(x,y,z\right)=\left(ax+\frac{1}{\left(a-c)(a-b\right)}i,by+\frac{1}{\left(b-a)(b-c\right)}i,cz+\frac{1}{\left(c-a)(c-b\right)}i\right).$$

We can reformulate then the system (12) in the form

(24) $$\left(\xi_{1}^{*}\left(t+\delta \right),\xi_{2}^{*}\left(t+\delta \right),\xi_{3}^{*}\left(t+\delta \right)\right)=S_{i}\left(\xi_{1}^{*}\left(t\right),\xi_{2}^{*}\left(t\right),\xi_{3}^{*}\left(t\right)\right),$$

where a,b,c∈(0,1),a≠b,b≠c,c≠a.

The family $\left\{S_{0},S_{1}:R^{3}\to R^{3}\right\}$ is an iterated function system if for every i∈I the mapping $S_{i}$ is a contraction on the complete Euclidean metric space $\left(R^{3},|\cdot |\right)$.

We can see that

(25) $$\begin{array}{c} |S_{0}\left(x,y,z\right)-S_{0}\left(x^{\prime },y^{\prime },z^{\prime }\right)\left|=\right|S_{1}\left(x,y,z\right)-S_{1}\left(x^{\prime },y^{\prime },z^{\prime }\right)|=\\ \sqrt{a^{2}{\left(x-x^{\prime }\right)}^{2}+b^{2}{\left(y-y^{\prime }\right)}^{2}+c^{2}{\left(z-z^{\prime }\right)}^{2}}\leq \\ \max\left\{a,b,c\right\}\sqrt{{\left(x-x^{\prime }\right)}^{2}+{\left(y-y^{\prime }\right)}^{2}+{\left(z-z^{\prime }\right)}^{2}}=\\ \max\left\{a,b,c\right\}|\left(x,y,z\right)-\left(x^{\prime },y^{\prime },z^{\prime }\right)|. \end{array}$$

Hence the mapping $S_{i}:R^{3}\to R^{3}$ is a contraction with the constant equal to max{a,b,c}<1.

**Definition** **2.** *Let $\left\{S_{i}:R^{3}\to R^{3}\;:\;i\in I\right\}$ be an iterated function system. We define the operator S on the set $A\subset R^{3}$ by the formula $S\left(A\right):=S_{0}\left(A\right)\cup S_{1}\left(A\right)$.*

The transformation S introduced above corresponds to the function (30) in the model from [17]. We will describe an invariant compact set *K* such that K=S(K).

**Remark** **2.** *In the paper [21] it was shown that for the metric space $R^{n}$ an iterated function system has a unique non-empty compact fixed set K such that $K=S\left(K\right)=S_{0}\left(K\right)\cup S_{1}\left(K\right).$ One way of generating such set K is to start with a compact set $A_{0}\subset R^{3}$ (which can contain a single point, called a seed) and iterate the mapping S using the formula $A_{n+1}=S\left(A_{n}\right)=S_{0}\left(A_{n}\right)\cup S_{1}\left(A_{n}\right)$. This iteration converges to the attractor $K=\lim_{n\to \infty }A_{n}$, i.e., the distance between K and $A_{n}$ converges to 0 in the Hausdorff metric, see [21].*

**Figure 3:**
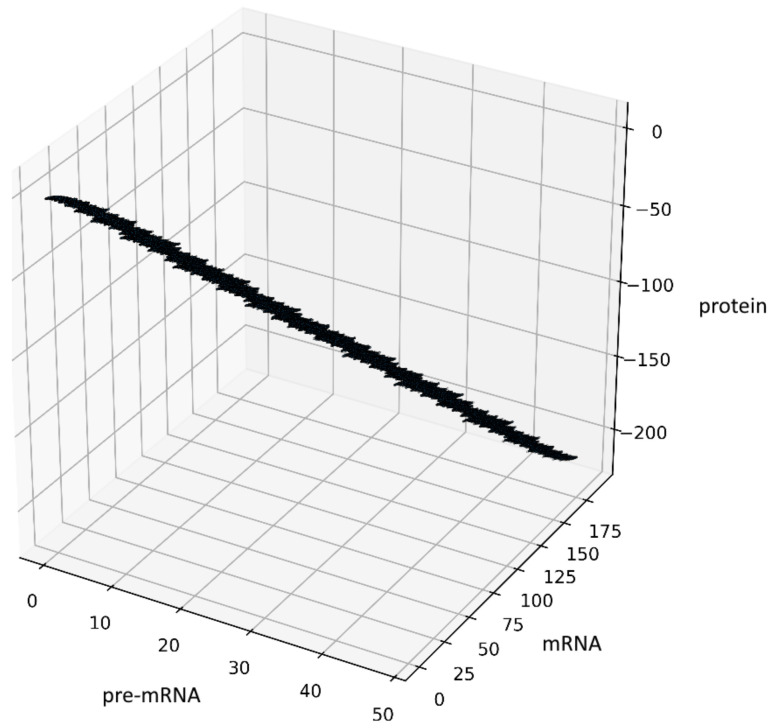
Figure presents the results obtained for the system (12) with $a=\frac{1}{2},b=\frac{3}{4}$, $c=\frac{2}{3},\delta =1$, the initial conditions $(\xi_{1}\left(0\right),\xi_{2}\left(0\right),\xi_{3}\left(0\right))=\left(0,0,0\right)$ and constant probabilities $p_{0}\left(x\right)\equiv 0.5,\;p_{1}\left(x\right)\equiv 0.5$ after 100,000 iterations.

Another way to generate some fractal objects was presented by Barnsley in [22]. The set of such points is called an IFS-attractor. In our case, an example of the attractor is shown in Figure 3, see also Section 10. The source code has been added to GitHub [19].

## 5. Iterated Function Systems with Place-Dependent Probabilities

In this section, similarly to the paper [23], we provide a description of IFS generated by the family of mappings $S_{0},S_{1}$ with $p_{i}\left(x\right),\;x\in R^{3},\;\;i\in \left\{0,1\right\}$ being a probability of a choice of a mapping $S_{i}$. We assume that $t\in Z_{\geq 0}$. Let $S_{0},S_{1}:R^{3}\to R^{3}$ be two Borel measurable non-singular functions, while let $p_{0}\left(x\right),\;p_{1}\left(x\right)$ be two non-negative Borel measurable functions such that $\forall_{x\in R^{3}}\;\;p_{0}\left(x\right)+p_{1}\left(x\right)=1$.

If $x\in R^{3}$ and $B\subset R^{3}$ is a Borel subset, then the transition probability from *x* to *B* is defined by

(26) $$P\left(x,B\right)=p_{0}\left(x\right)1\;l_{B}\left(S_{0}\left(x\right)\right)+p_{1}\left(x\right)1\;l_{B}\left(S_{1}\left(x\right)\right),$$

where $1\;l_{B}$ is the indicator function of the set *B*. We can define the mapping

$$\left(Tg\right)\left(x\right)=\int_{R^{3}}g\left(y\right)P\left(x,dy\right)=p_{0}\left(x\right)g\left(S_{0}\left(x\right)\right)+p_{1}\left(x\right)g\left(S_{1}\left(x\right)\right),$$

where *T* is a Markov operator on space of the bounded Borel measurable real-valued functions (which forms the Banach space with the supremum norm). Then $T1\;l_{B}=P\left(x,B\right)$. Let $M\left(R^{3}\right)=\left\{\nu |\;\;\nu :B\left(R^{3}\right)\to R,\;\;\nu \left(\emptyset \right)=0,\;\;\nu \;\;\mathrm{is}\;\sigma -\mathrm{additive}\right\}$ be the space of finite signed Borel measures on $R^{3}$. By $P\left(R^{3}\right)\subset M\left(R^{3}\right)$ we denote the set of all probability measures from $M(R^{3})$.

We define the operator $F:M\left(R^{3}\right)∋\nu \to F_{\nu }\in M\left(R^{3}\right)\;\;$ by the formula

(27) $$\begin{array}{c} F_{\nu }\left(B\right)=\int_{R^{3}}P\left(x,B\right)d\nu \left(x\right)=\int_{S_{0}^{-1}\left(B\right)}p_{0}\left(x\right)d\nu \left(x\right)+\int_{S_{1}^{-1}\left(B\right)}p_{1}\left(x\right)d\nu \left(x\right)=\\ \int_{B}P_{S_{0}}p_{0}\left(x\right)d\nu \left(x\right)+\int_{B}P_{S_{1}}p_{1}\left(x\right)d\nu \left(x\right), \end{array}$$

showing how a probability distribution ν on *X* of the process is transformed in one step. Here, the operators $P_{S_{0}},\;P_{S_{1}}$ are classical Frobenius–Perron operators for the transformation $S_{0},S_{1}$, respectively (see [20], Section 2.1.5). Let $C(R^{3})$ be the set of bounded real-valued continuous functions on $R^{3}$. A Borel invariant probability measure μ (i.e., Fμ=μ) is called attractive iff for all $\nu \in P(R^{3})$ and for all $f\in C(R^{3})$ we have $\lim_{n\to \infty }\int fd\left(F^{n}\nu \right)=\int fd\mu$. In other words, that means $F^{n}\nu$ converges to μ in distribution. For the rest of the section, we will use the theory of Markov processes (see p. 369 in [18]) to describe this IFS. Let $\left(X_{t}^{\mu }\right)$ be the Markov process with initial distribution equal to $\mu \in P(R^{3})$ and transition probability P(x,B) from point *x* to Borel subset $B\subset R^{3}$. If μ is a Dirac measure concentrated at $x_{0}$, then we denote the process $\left(X_{t}^{x_{0}}\right)$. A transition probability *P* provides the following interpretation. We have $P\left(x,B\right)=P\left(X_{1}^{x_{0}}\in B|X_{0}^{x_{0}}=x\right)$. If $X_{0}^{\mu }$ has a distribution $\mu \in P(R^{3})$, then $F_{\mu }$ is the distribution of $X_{1}^{\mu }$ which means that $P\left(X_{1}^{\mu }\in B\right)=F_{\mu }\left(B\right)$. It is known that

$$Tf\left(x_{0}\right)=\int_{R^{3}}f\left(y\right)P\left(x_{0},dy\right)=E\left(f\left(X_{1}^{x_{0}}\right)|X_{0}^{x_{0}}=x_{0}\right)=Ef\left(X_{1}^{x_{0}}\right),$$

where *f* is a bounded Borel measurable real-valued function.

Hence, $Ef\left(X_{1}^{x_{0}}\right)=p_{0}\left(x\right)f\left(S_{0}\left(x\right)\right)+p_{1}\left(x\right)f\left(S_{1}\left(x\right)\right)$. In the next section we investigate long-term behavior of the process $\left(X_{t}^{\mu }\right)$.

## 6. Convergence of the System to Invariant Measure

In this section we assume that $t\in Z_{\geq 0}$. In classical work [18], Barnsley considered a discrete-time Markov process $\left(X_{t}^{\mu }\right)$ on a locally compact metric space *X* obtained by a family of randomly iterating Lipschitz maps $S_{0},\;\ldots ,\;S_{n},\;n\in N$. For any *i* the probability of choosing map $S_{i}$ at each step is given by $p_{i}\left(x\right)$. Assume that:

1. Sets of finite diameter in *X* have compact closure.
2. For any *i* the mappings $S_{i}$ are average-contractive, i.e., $\sum_{i=1}^{N}p_{i}\left(x\right)\;\log\frac{d(S_{i}\left(x\right),S_{i}\left(y\right))}{d(x,y)}<0,$ uniformly in *x* and *y*, (for details see paper [18]).
3. $\exists_{\delta_{0}>0}\;\forall_{x\in X}\;\;p_{i}\left(x\right)\geq \delta_{0}.$
4. For every *i* the mappings $p_{i}\left(x\right)$ are Hölder continuous.

Under these assumptions, the Markov process $\left(X_{t}\right)$ converges in distribution to a unique invariant measure. In our regime, we can formulate a weaker version of the theorem above (see also [18], p. 372).

**Theorem** **1.** *Let $\left(X_{t}^{\mu }\right)$ be a Markov process on the space $R^{3}\times \left\{0,1\right\}$. We assume that the initial distribution of this process is given by $\mu \in P(R^{3})$ and its transition probability is given by (26). Let the probability $p_{i}\left(x\right)$ of choosing contractive map $S_{i}$ at each step be Hölder continuous function and moreover*

(28) $$\exists_{\delta_{0}>0}\;\forall_{x\in R^{3}}\;p_{i}\left(x\right)\geq \delta_{0}.$$

*Then the Markov process $\left(X_{t}\right)$ converges in distribution to a unique invariant measure when t→∞.*

To illustrate this theorem, we will investigate transition probability in the case of the stochastic process $\left(X_{t}\right)$, see (22). We assume that the state space of our process is $R^{3}\times \left\{0,1\right\}$. For j∈{0,1} we define the jump transformation $R_{j}:R^{3}\times \left\{0,1\right\}\to R^{3}\times \left\{0,1\right\}$ by the formula $R_{j}\left(x,i\right)=\left(x,j\right)$.

Each jump transformation $R_{0},R_{1}$, defined on the state space $R^{3}\times \left\{0,1\right\}$ is non-singular with respect to the product measure μ of the Lebesgue measure on $R^{3}$ and the counting measure on the set {0,1}. We define the positive and continuous jump intensity rate functions by the formulas $q_{0}=q_{0}\left(\xi_{1},\xi_{2},\xi_{3}\right)$ and $q_{1}=q_{1}\left(\xi_{1},\xi_{2},\xi_{3}\right)$ on $R^{3}$. Here, $q_{i}$ is the jump intensity rate from the state *i* to the state 1−i, where i∈{0,1} see Figure 1. Let $p_{j}\left(x,i\right)=p_{j}\left(x\right)$. The following equation holds:

(29) $$P\left(\left(x,i\right),\left\{\left(x,j\right)\right\}\right)=P\left(X_{1}=\left(x,j\right)|X_{0}=\left(x,i\right)\right)=p_{j}\left(x\right).$$

Please note that $P\left(\left(x,0\right),\left\{\left(x,j\right)\right\}\right)=P\left(\left(x,1\right),\left\{\left(x,j\right)\right\}\right)=p_{j}\left(x\right)$.

Let $S_{0},S_{1}:R^{3}\to R^{3}$ be two Borel measurable non-singular functions. If $x\in R^{3}$ and $B\subset R^{3}$ is a Borel subset, then the transition probability is defined by:

(30) $$\begin{array}{c} P\left(\left(x,i\right),B\times \left\{0,1\right\}\right)=P\left(X_{1}\in B\times \left\{0,1\right\}|X_{0}=\left(x,i\right)\right)=\\ p_{0}\left(x\right)1\;l_{B}\left(S_{0}\left(x\right)\right)+p_{1}\left(x\right)1\;l_{B}\left(S_{1}\left(x\right)\right)>\delta_{0}. \end{array}$$

Please note that $X_{1}\in \left\{\left(S_{0}\left(x\right),0\right),\left(S_{1}\left(x\right),1\right)\right\}$.

Assume now that the initial distribution of the process $\left(X_{t}\right)$ is given by $\mu \in P(R^{3})$ and its transition probability is given by (30). The process $\left(X_{t}\right)$ is both Markov and IFS such that the probability of random choice of one of two functions $S_{0},S_{1}$ depends on the space part of a state. By Theorem 1 the Markov process $\left(X_{t}\right)$ converges in distribution to a unique invariant measure when t→∞.

## 7. Properties of an Invariant Measure

In this section we assume that $t\in Z_{\geq 0}$ (for the sake of simplicity, we assume here that δ=1). By Theorem 1, we know that the process $\left(X_{t}\right)$ converges in distribution to a unique invariant measure. A classical result of Hutchinson [21] states that there exists a unique non-empty compact set *K* such that $K=S\left(K\right)=S_{0}\left(K\right)\cup S_{1}\left(K\right)$.

**Theorem** **2.** *Consider the stochastic process $\left(X_{t}^{\mu }\right)$ such that $X_{0}^{\mu }=x\in R^{3}$ with $\mu \in P(R^{3})$ and transition probability given by (30). Then*

(31) $$\inf\{d\left(X_{t}^{\mu },y\right):y\in K\}\to 0,$$

*where d is the Euclidean distance in $R^{3}$.*

**Proof.**  For any set $A\subset R^{3}$ we denote $S^{0}\left(A\right)=A,S^{1}\left(A\right)=S\left(A\right)$ and $S^{p}\left(A\right)=S\left(S^{p-1}\left(A\right)\right)$ for p≥2. Consider A={x}. From the Theorem 3.1 (Ch. 3, p. viii) of [21] it follows then $S^{p}\left(A\right)$ converges to *K* in the Hausdorff metric uniformly when p→∞. Using our notion, $\inf\{d\left(S_{i_{0}}\circ S_{i_{1}}\circ \ldots S_{i_{t}}\left(x\right),y\right):y\in K\}$ converges to 0 uniformly when t→∞. Please note that $S_{i_{0}}\circ S_{i_{1}}\circ \ldots S_{i_{t}}\left(x\right)$ is a trajectory of our process which depends on the probabilities $p_{0},p_{1}$, see (30). Hence, we get $\inf\{d\left(X_{t}^{\mu },y\right):y\in K\}\to 0$, since the choice of $x\in R^{3}$ was arbitrary.□

Moreover, if $X_{t}^{\mu }\in K$, then $X_{t+1}^{\mu }\in \left\{S_{0}\left(X_{t}^{\mu }\right),S_{1}\left(X_{t}^{\mu }\right)\right\}\subset S_{0}\left(K\right)\cup S_{1}\left(K\right)=K$. Hence, *K* is an invariant set for this process.

## 8. Jump Distribution

**Remark** **3.** *Let t∈[0,∞). With an analogy to the description of PDMP in the book [14], we will define the function $F_{\xi ,i}$ as a cumulative distribution function of the first jump $t_{1}$ of the process $\left(X_{t}\right)$ which starts at t=0 at some point $\left(\xi ,i\right)\in R^{3}\times \left\{0,1\right\}$. Let $F_{\xi ,i}\left(t\right):=Prob\left\{t_{1}\leq t\right\}$ and we define then the process on the random interval $\left[0,t_{1}\right]$ as follows:*

(32) $$X_{t}=\left\{\begin{array}{cc} (\pi^{i}\left(t,\xi \right),i), & t<t_{1};\\ \left(\pi^{i}\left(t,\xi \right),1-i\right) & t=t_{1}. \end{array}\right.$$

*After time $t_{1}$ the process $\left(X_{t}\right)$ starts again, but with new initial condition equal to $X(t_{1})$.*

*This process evolves with respect to the points obtained by the solution (12) with given value of i until time of the next jump $t_{2}$. Then, this step repeats infinitely many times. Please note that*

$$Prob\left(t_{1}>t\right)=1-F_{\xi ,i}\left(t\right)=\exp\left\{-\sum_{s=0}^{\lfloor t\rfloor -1}q_{i}\left(\pi^{i}\left(s,\xi \right)\right)\right\}.$$

*Hence, $Prob(t_{1}>t)>0$ for all t≥0, because $q_{i}$ is a bounded function.*

*Also, $Prob(t_{1}>t)<1$ for all t≥1, because $q_{i}$ is positive function.*

*Hence, $t_{1}>0$. Analogically, $\Delta t_{k-1}=t_{k}-t_{k-1}>0$ for all k≥1, where $t_{0}=0$. All these considerations are true with the probability being equal to 1.*

*Let $\mu =\max_{y\in {\left[0,2\right]}^{3},i\in \left\{0,1\right\}}q_{i}\left(y\right)$. Next, by (20) we get $F_{\xi ,i}\left(t\right)\leq 1-exp\left\{-\mu t\right\}$ for all $y\in {\left[0,2\right]}^{3}$. Please note that $Prob\left(\Delta t_{k-1}\right)\leq 1-\exp\left\{-\mu t\right\},$ independently from the values of $\Delta_{i}$, where 0≤i≤k−2. Hence, $Prob\left(T_{k}\leq t\right)\leq {\left(1-\exp\left(-\mu t\right)\right)}^{k}$.*

*Therefore, $\lim_{k\to \infty }T_{k}=\infty$. We also get that*

(33) $$\begin{array}{c} E\left(\max\left\{k\geq 0,T_{k}<t\right\}\right)=\sum_{k=0}^{\infty }Prob\left(N_{t}\geq k\right)=\\ \sum_{k=0}^{\infty }Prob\left(T_{k}<t\right)\leq \sum_{k=0}^{\infty }{\left(1-\exp\left(-\mu t\right)\right)}^{k}=\exp\left(\mu t\right)<\infty , \end{array}$$

*where t>0. Please note that $E(\max\left\{k\geq 0,T_{k}<t\right\})$ is the expected value of the number of jumps of our process up to the time t.*

Now we will gather all the facts about the process $\left(X_{t}\right)$ considered in this paper.

**Definition of the process**

**1.**  Denote the state space $R^{3}\times \left\{0,1\right\}$.
**2.**  According to the reaction scheme Figure 1, the reactions which occur in our process are as follows:
  $$\mathrm{Inactive}\overset{q_{0}\left(\xi_{1},\xi_{2},\xi_{3}\right)}{\to }\mathrm{Active},\;\mathrm{Inactive}\overset{q_{1}\left(\xi_{1},\xi_{2},\xi_{3}\right)}{\leftarrow }\mathrm{Active},$$
  **Outcome (A)**
  $$\begin{array}{c} Active\overset{R}{\underset{\mathrm{with}\;\mathrm{probability}\;p_{0}\left(\xi_{1},\xi_{2},\xi_{3}\right)}{\to }}\mathrm{pre}-\mathrm{mRNA}\overset{C+\mu_{PR}}{\to }\\ \overset{C+\mu_{PR}}{\to }\mathrm{Degeneration}\;\mathrm{or}\;\mathrm{pre}-\mathrm{mRNA}\;\mathrm{conversion}\;\mathrm{to}\;\mathrm{mRNA}, \end{array}$$
  **Outcome (B)**
  $$\begin{array}{c} Inactive\overset{0}{\underset{\mathrm{with}\;\mathrm{probability}\;p_{1}\left(\xi_{1},\xi_{2},\xi_{3}\right)}{\to }}\mathrm{pre}-\mathrm{mRNA}\overset{C+\mu_{PR}}{\to }\\ \overset{C+\mu_{PR}}{\to }\mathrm{Degeneration}\;\mathrm{or}\;\mathrm{pre}-\mathrm{mRNA}\;\mathrm{conversion}\;\mathrm{to}\;\mathrm{mRNA}, \end{array}$$
  **Outcome (A) and (B)**
  $$\begin{array}{c} \mathrm{pre}-\mathrm{mRNA}\overset{C}{\to }\mathrm{mRNA}\overset{\mu_{R}}{\to }\mathrm{Degeneration},\\ \mathrm{mRNA}\overset{P}{\to }\mathrm{Protein}\overset{\mu_{P}}{\to }\mathrm{Degeneration}, \end{array}$$
  where $\left(\xi_{1}\left(t\right),\xi_{2}\left(t\right),\xi_{3}\left(t\right)\right)$ is the concentration level of all the substances at time *t*.
  Consider the simplified version of this system (12) with $S_{0},S_{1}:R^{3}\to R^{3}$ being two Borel measurable non-singular functions defined by (23).
**3.**  Let $p_{0}\left(x\right),p_{1}\left(x\right)$ be two non-negative Borel measurable functions such that
  $$\forall_{x\in R^{3}}\;\;p_{0}\left(x\right)+p_{1}\left(x\right)=1.$$
**4.**  In addition, let $\mu \in P(R^{3})$ and $q_{0}$ and $q_{1}$ be two non-negative functions defined on $R^{3}$.
**5.**  From now, by $\pi^{i}\left(t,\bar{\xi }\right)$ we denote the solutions of the system (12), i.e.,
  (34) $$\pi^{i}\left(t,\bar{\xi }\right)=\left(\xi_{1}^{*}\left(t\right),\xi_{2}^{*}\left(t\right),\xi_{3}^{*}\left(t\right)\right).$$
  Despite the fact that we consider discrete-time Markov process, we can assume that t∈[0,∞) (see comment above Equation (8)). We consider two cases, where i=0 or i=1, which corresponds to the functions $S_{0}$ and $S_{1},$ respectively.
**6.**  Let ${\left(X_{n}^{\mu }\right)}_{n=0}^{\infty }$ be a Markov process on the space $R^{3}\times \left\{0,1\right\}$ with initial distribution of the process given by $\mu \in P(R^{3})$ and its transition probability is given by (30).
**7.**  Here, $X_{1}\in \left\{\left(S_{0}\left(x\right),0\right),\left(S_{1}\left(x\right),1\right)\right\}$.
**8.**  ${\left(X_{n}^{\mu }\right)}_{n=0}^{\infty }$ is both Markov process and IFS such that the probability of random choice of one of two functions $S_{0},S_{1}$ depends on the space part of a state.
**9.**  With an analogy to the description in the book [14], we define the function $F_{\xi ,i}$ as a cumulative distribution function of the first jump $t_{1}$ of our process $\left(X_{t}\right)$ which starts at t=0 at some point $\left(\xi ,i\right)\in R^{3}\times \left\{0,1\right\}$.
**10.**  We say that $Prob\left(t_{1}\leq t\right)=F_{\xi ,i}\left(t\right)$ and we define then the process on the random interval $\left[0,t_{1}\right]$ as follows:
  (35) $$X_{t}=\left\{\begin{array}{cc} (\pi^{i}\left(t,\xi \right),i), & t<t_{1};\\ \left(\pi^{i}\left(t,\xi \right),1-i\right) & t=t_{1}. \end{array}\right.$$
**11.**  After time $t_{1}$ we start the process *X* again, but with new initial conditions being equal to $X(t_{1})$. This process evolves with respect to the points obtained by the solution (12) with given value of *i* till time of the next jump $t_{2}$. Then, we repeat this step infinitely many times. Since $Prob\left(t_{1}>t\right)=1-F_{\xi ,i}\left(t\right)=\exp\left\{-\sum_{s=0}^{\lfloor t\rfloor -1}q_{i}\left(\pi^{i}\left(s,\xi \right)\right)\right\}$.
**12.**  From the definition of the process $\left(X_{t}\right)$ both of the intensity functions $q_{0}$ and $q_{1}$ depend on two non-negative Borel measurable functions $p_{0}\left(x\right),p_{1}\left(x\right)$.

**Summary of the properties of the process**$\left(X_{t}\right)$ is both Markov process and IFS such that the probability of random choice of one of two functions $S_{0},S_{1}$ depends on the space part of a state. By Theorem 1 the Markov process $\left(X_{t}\right)$ converges in distribution to a unique invariant measure when n→∞. This theorem means that the trajectories of this process after sufficiently long time are arbitrarily close to *K* independent from the probability distribution. In addition, if $X_{n}^{\mu }\in K$, then $X_{n+1}^{\mu }\in \left\{S_{0}\left(X_{n}^{\mu }\right),S_{1}\left(X_{n}^{\mu }\right)\right\}\subset S_{0}\left(K\right)\cup S_{1}\left(K\right)=K$. Hence, *K* is invariant. It is worth noting that the life-span function of the process is equal to $\exp\{-\sum_{s=0}^{\lfloor t\rfloor -1}q\left(\pi \left(s,x_{0}\right)\right)\},$ unlike the continuous case studied in [17].

## 9. Stochastic Simulations

**Figure 4:**
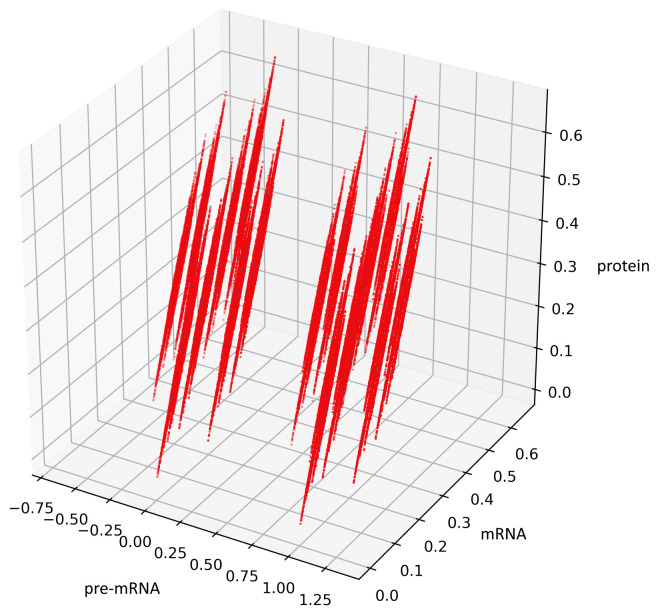
Visualization of the stochastic process (4), depending on two non-negative Borel measurable functions $p_{0}\left(x\right)=\frac{1}{2(1+|x{|}^{2})}$ and $p_{1}\left(x\right)=1-p_{0}\left(x\right)=\frac{1+{2|x|}^{2}}{2(1+|x{|}^{2})}$.

To visualize the behavior of the stochastic process (4), we performed stochastic simulation of the process (Figure 4). The code was developed in Python (3.7.4). The parameter values are $\delta =1,R=1,\mu_{PR}=\frac{1}{4},\;C=\mu_{R}=\frac{1}{4},P=\mu_{P}=\frac{1}{3}$ with Borel measurable probability functions $p_{0}\left(x\right)=\frac{1}{2(1+|x{|}^{2})}$ and $p_{1}\left(x\right)=1-p_{0}\left(x\right)=\frac{1+{2|x|}^{2}}{2(1+|x{|}^{2})}$ and initial conditions $x\left(0\right)=y\left(0\right)=z\left(0\right)=\frac{1}{2}$.

## 10. The Derivation of the Formula for the Attractor

We consider the system which simplifies both systems (1) and (6), namely (12):

(36) $$\begin{array}{ccc} \xi_{1}^{*}\left(t+\delta \right) & = & a\xi_{1}^{*}\left(t\right)+\frac{1}{\left(a-c)(a-b\right)}i\\ \xi_{2}^{*}\left(t+\delta \right) & = & b\xi_{2}^{*}\left(t\right)+\frac{1}{\left(b-a)(b-c\right)}i\\ \xi_{3}^{*}\left(t+\delta \right) & = & c\xi_{3}^{*}\left(t\right)+\frac{1}{\left(c-a)(c-b\right)}i, \end{array}$$

with the initial condition $\bar{\xi }=\left(\xi_{1}\left(0\right),\xi_{2}\left(0\right),\xi_{3}\left(0\right)\right)\in R^{3}$. We also assume that the values of the parameters a,b,c∈(0,1) are pairwise distinct.

In the case of δ=1, we will find a set for the process described by the system (36), i.e., the smallest invariant set for the process, for which almost all trajectories of the process enter in a finite time.

**Remark** **4.** *Let us observe that if we consider only integer values of t then the attractor generated by the composition of the systems (36) is a discrete set (see Figure 3 for $a=\frac{1}{2}$, $b=\frac{3}{4},\;c=\frac{2}{3}$) and it is contained inside the attractor obtained for real values t. Hence, now we only proceed with real values of t.*

Let $x=(x_{1},x_{2},x_{3})$. Let $\pi_{t}^{i}\left(x\right)=\left(\xi_{1}^{*}\left(t\right),\xi_{2}^{*}\left(t\right),\xi_{3}^{*}\left(t\right)\right)$ denote the solutions of (36) at time *t* with the initial condition x. Namely

$$\pi_{t}^{i}\left(x\right)=\left(a^{t}\left(x_{1}-v_{1}i\right)+v_{1}i,b^{t}\left(x_{2}-v_{2}i\right)+v_{2}i,c^{t}\left(x_{3}-v_{3}i\right)+v_{3}i\right),$$

where by v we denote the vector

(37) $$\left(v_{1},v_{2},v_{3}\right)=\left(\frac{1}{\left(a-c)(a-b)(1-a\right)},\frac{1}{\left(b-a)(b-c)(1-b\right)},\frac{1}{\left(c-a)(c-b)(1-c\right)}\right).$$

We obtain

(38) $$\begin{array}{ccc} \pi_{t}^{0}\left(x\right) & = & (a^{t}x_{1},b^{t}x_{2},c^{t}x_{3}),\\ \pi_{t}^{1}\left(x\right) & = & v+\pi_{t}^{0}\left(x\right)-\pi_{t}^{0}\left(v\right). \end{array}$$

This gives us the following formulas:

$$\begin{array}{ccc} \pi_{t_{2}}^{1}\pi_{t_{1}}^{0}\left(x\right) & = & v+\pi_{t_{1}+t_{2}}^{0}\left(x\right)-\pi_{t_{2}}^{0}\left(v\right),\\ \pi_{t_{2}}^{0}\pi_{t_{1}}^{1}\left(x\right) & = & \pi_{t_{2}}^{0}\left(v\right)+\pi_{t_{1}+t_{2}}^{0}\left(x\right)-\pi_{t_{1}+t_{2}}^{0}\left(v\right) \end{array}$$

for all times $t_{1},t_{2}\geq 0$. Hence

$$\begin{array}{ccc} \pi_{t_{3}}^{0}\pi_{t_{2}}^{1}\pi_{t_{1}}^{0}\left(x\right) & = & \pi_{t_{3}}^{0}\left(v\right)+\pi_{t_{1}+t_{2}+t_{3}}^{0}\left(x\right)-\pi_{t_{2}+t_{3}}^{0}\left(v\right),\\ \pi_{t_{3}}^{1}\pi_{t_{2}}^{0}\pi_{t_{1}}^{1}\left(x\right) & = & v+\pi_{t_{2}+t_{3}}^{0}\left(v\right)+\pi_{t_{1}+t_{2}+t_{3}}^{0}\left(x\right)-\pi_{t_{1}+t_{2}+t_{3}}^{0}\left(v\right)-\pi_{t_{3}}^{0}\left(v\right). \end{array}$$

Using the formulas (38) we get

(39) $$\begin{array}{c} \pi_{t_{2}}^{0}\pi_{t_{1}}^{1}\left(x\right)=\\ (a^{t_{2}}v_{1}+a^{t_{1}+t_{2}}\left(x_{1}-v_{1}\right),b^{t_{2}}v_{2}+b^{t_{1}+t_{2}}\left(x_{2}-v_{2}\right),c^{t_{2}}v_{3}+c^{t_{1}+t_{2}}\left(x_{3}-v_{3}\right)), \end{array}$$

(40) $$\begin{array}{c} \pi_{t_{2}}^{1}\pi_{t_{1}}^{0}\left(x\right)=\\ (v_{1}-a^{t_{2}}v_{1}+a^{t_{1}+t_{2}}x_{1},v_{2}-b^{t_{2}}v_{2}+b^{t_{1}+t_{2}}x_{2},v_{3}-c^{t_{2}}v_{3}+c^{t_{1}+t_{2}}x_{3}). \end{array}$$

If $t_{1},t_{2}\in \left[0,\infty \right)$, we can assume $\alpha :=a^{t_{2}},\;\beta :=a^{t_{1}+t_{2}}$. Hence,

(41) $$\begin{array}{c} \pi_{t_{2}}^{0}\pi_{t_{1}}^{1}\left(x\right)=\\ (\alpha v_{1}+\beta \left(x_{1}-v_{1}\right),\alpha^{\log_{a}b}v_{2}+\beta^{\log_{a}b}\left(x_{2}-v_{2}\right),\alpha^{\log_{a}c}v_{3}+\beta^{\log_{a}c}\left(x_{3}-v_{3}\right)), \end{array}$$

(42) $$\begin{array}{c} \pi_{t_{2}}^{1}\pi_{t_{1}}^{0}\left(x\right)=\\ (v_{1}-\alpha v_{1}+\beta x_{1},v_{2}-\alpha^{\log_{a}b}v_{2}+\beta^{\log_{a}b}x_{2},v_{3}-\alpha^{\log_{a}c}v_{3}+\beta^{\log_{a}c}x_{3}), \end{array}$$

where 1≥α≥β>0. These equations are similar to the ones obtained in the continuous case [17], therefore the attractor will adopt analogous form as in that case.

Taking as the initial points x=(0,0,0) in the Formula (41) and $x=(v_{1},v_{2},v_{3})$ in the Formula (42), we get a parametric equations for the surfaces $A_{0}$ and $A_{1}$ which we will found out as the boundaries of attractor A:

$$\begin{array}{ccc} A_{0} & = & \{\left(\alpha -\beta \right)v_{1},\left(\alpha^{\log_{a}b}-\beta^{\log_{a}b}\right)v_{2},\left(\alpha^{\log_{a}c}-\beta^{\log_{a}c}\right)v_{3})\},\\ A_{1} & = & \{\left(v_{1}\left(1-\alpha +\beta \right),v_{2}\left(1-\alpha^{\log_{a}b}+\beta^{\log_{a}b}\right),v_{3}\left(1-\alpha^{\log_{a}c}+\beta^{\log_{a}c}\right)\right)\}. \end{array}$$

Please note that both sets are symmetric to each other with respect to the point $\left(\frac{v_{1}}{2},\frac{v_{2}}{2},\frac{v_{3}}{2}\right)$, since $\left(v_{1},v_{2},v_{3}\right)-A_{0}=A_{1}$. This means that the boundary of A (and so is the attractor A) is symmetric to itself with respect to the point $\left(\frac{v_{1}}{2},\frac{v_{2}}{2},\frac{v_{3}}{2}\right)$. Moreover, it can be shown that

(43) $$(v_{1},v_{2},v_{3})-A=A.$$

Now we are going to describe the attractor A. It appears that two changes of *i* are sufficient to get to any arbitrary point in A. The composition of three flows $\pi_{t_{3}}^{1}\pi_{t_{2}}^{0}\pi_{t_{1}}^{1}$ and $\pi_{t_{3}}^{0}\pi_{t_{2}}^{1}\pi_{t_{1}}^{0}$ is given by the following formulas:

(44) $$\begin{array}{ccc} \pi_{t_{3}}^{1}\pi_{t_{2}}^{0}\pi_{t_{1}}^{1}\left(x\right) & = & (v_{1}\left(1-a^{t_{3}}+a^{t_{2}+t_{3}}-a^{t_{1}+t_{2}+t_{3}}\right)+a^{t_{1}+t_{2}+t_{3}}x_{1},\\ & & v_{2}\left(1-b^{t_{3}}+b^{t_{2}+t_{3}}-b^{t_{1}+t_{2}+t_{3}}\right)+b^{t_{1}+t_{2}+t_{3}}x_{2},\\ & & v_{3}\left(1-c^{t_{3}}+c^{t_{2}+t_{3}}-c^{t_{1}+t_{2}+t_{3}}\right)+c^{t_{1}+t_{2}+t_{3}}x_{3}), \end{array}$$

(45) $$\begin{array}{ccc} \pi_{t_{3}}^{0}\pi_{t_{2}}^{1}\pi_{t_{1}}^{0}\left(x\right) & = & (v_{1}\left(a^{t_{3}}-a^{t_{2}+t_{3}}\right)+a^{t_{1}+t_{2}+t_{3}}x_{1},\\ & & v_{2}\left(b^{t_{3}}-b^{t_{2}+t_{3}}\right)+b^{t_{1}+t_{2}+t_{3}}x_{2},\\ & & v_{3}\left(c^{t_{3}}-c^{t_{2}+t_{3}}\right)+c^{t_{1}+t_{2}+t_{3}}x_{3}). \end{array}$$

**Figure 5:**
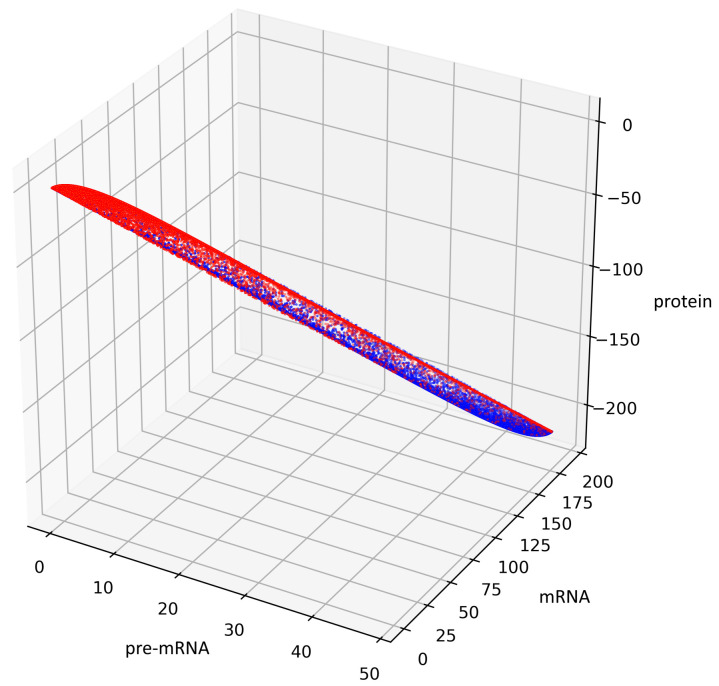
This figure presents all states of the stochastic process (36) with $a=\frac{1}{2},\;b=\frac{3}{4},\;c=\frac{2}{3}$ after two switches. The red color represents the states given by the Formula (45) representing trajectories of the process starting with i=0 while the blue color represents the states given by the Formula (44) representing trajectories of the process starting with i=1.

Figure 5 presents trajectories of the processes (44) and (45), where $t_{1},t_{2},t_{3}$ are drawn from uniform distribution on the interval (0,100). Both show the contour of the attractor A. For parameters chosen to create Figure 5, the density of colors intensity (i.e., red intensity, blue intensity) and Equations (39) and (40) may suggest bistability (in the sense of bimodality of the stationary distribution, see [17]). We are convinced that there is a need for further research about bistability in a discrete case. Please note that for deterministic linear systems, bistability cannot hold, hence such phenomenon in a stochastic linear system would be interesting.

We will start with description of the set, which we can reach in two changes of i. In analogy to the above, in the case of double superposition, we define α,β,γ in a new way. If $t_{1},t_{2},t_{3}\in \left[0,\infty \right)$, we can assume $\alpha :=a^{t_{3}},\;\beta :=a^{t_{2}+t_{3}},\;\gamma :=a^{t_{1}+t_{2}+t_{3}}$ and hence we get equations:

(46) $$\begin{array}{ccc} \pi_{t_{3}}^{1}\pi_{t_{2}}^{0}\pi_{t_{1}}^{1}\left(x\right) & = & (v_{1}\left(1-\alpha +\beta -\gamma \right)+\gamma x_{1},\\ & & v_{2}\left(1-\alpha^{\log_{a}b}+\beta^{\log_{a}b}-\gamma^{\log_{a}b}\right)+\gamma^{\log_{a}b}x_{2},\\ & & v_{3}\left(1-\alpha^{\log_{a}c}+\beta^{\log_{a}c}-\gamma^{\log_{a}b}\right)+\gamma^{\log_{a}c}x_{3}), \end{array}$$

(47) $$\begin{array}{ccc} \pi_{t_{3}}^{0}\pi_{t_{2}}^{1}\pi_{t_{1}}^{0}\left(x\right) & = & (v_{1}\left(\alpha -\beta \right)+\gamma x_{1},\\ & & v_{2}\left(\alpha^{\log_{a}b}-\beta^{\log_{a}b}\right)+\gamma^{\log_{a}b}x_{2},\\ & & v_{3}\left(\alpha^{\log_{a}c}-\beta^{\log_{a}c}\right)+\gamma^{\log_{a}c}x_{3}), \end{array}$$

where 1≥α≥β≥γ>0 and

$$\left(v_{1},v_{2},v_{3}\right)=\left(\frac{1}{\left(a-c)(a-b)(1-a\right)},\frac{1}{\left(b-a)(b-c)(1-b\right)},\frac{1}{\left(c-a)(c-b)(1-c\right)}\right).$$

We can assume that 1≥α≥β≥γ≥0 because if γ=0 in Equations (46) and (47) then we get:

$$\begin{array}{c} \pi_{t_{3}}^{1}\pi_{t_{2}}^{0}\pi_{t_{1}}^{1}\left(x\right)=\left(v_{1}\left(1-\alpha +\beta \right),v_{2}\left(1-\alpha^{\log_{a}b}+\beta^{\log_{a}b}\right),v_{3}\left(1-\alpha^{\log_{a}c}+\beta^{\log_{a}c}\right)\right), \end{array}$$

$$\begin{array}{ccc} \pi_{t_{3}}^{0}\pi_{t_{2}}^{1}\pi_{t_{1}}^{0}\left(x\right) & = & (v_{1}\left(\alpha -\beta \right),v_{2}\left(\alpha^{\log_{a}b}-\beta^{\log_{a}b}\right),v_{3}\left(\alpha^{\log_{a}c}-\beta^{\log_{a}c}\right)), \end{array}$$

(the values of above states can be also obtained taking γ>0 and after appropriate substitution to $\alpha ,\beta ,(x_{1},x_{2},x_{3})$). Please note that these states belong correspondingly to the boundaries $A_{1}$ and $A_{0}$. Hence γ=0 is a case when the trajectory is on the boundary of the attractor A.

Let

(48) $$\begin{array}{c} A=\{\left(\varphi_{a,b,c}\left(x,y,z\right),\chi_{a,b,c}\left(x,y,z\right),\psi_{a,b,c}\left(x,y,z\right)\right):1\geq x\geq y\geq z\geq 0\}, \end{array}$$

where

(49) $$\begin{array}{ccc} \varphi_{a,b,c}\left(x,y,z\right) & := & \frac{1}{\left(a-c)(a-b)(1-a\right)}\left(x-y+z\right),\\ \chi_{a,b,c}\left(x,y,z\right) & := & \frac{1}{\left(b-a)(b-c)(1-b\right)}\left(x^{\log_{a}b}-y^{\log_{a}b}+z^{\log_{a}b}\right),\\ \psi_{a,b,c}\left(x,y,z\right) & := & \frac{1}{\left(c-a)(c-b)(1-c\right)}\left(x^{\log_{a}c}-y^{\log_{a}c}+z^{\log_{a}c}\right). \end{array}$$

The set A consist of all points from (47), where we take $\left(x_{1},x_{2},x_{3}\right)=\left(v_{1},v_{2},v_{3}\right)$.

Equivalently using the Equation (46) we get an alternative formula for the set A.

(50) $$A=\{\left(\varphi_{a,b,c}^{\prime }\left(x,y,z\right),\chi_{a,b,c}^{\prime }\left(x,y,z\right),\psi_{a,b,c}^{\prime }\left(x,y,z\right)\right):1\geq x\geq y\geq z\geq 0\},$$

where

(51) $$\begin{array}{ccc} \varphi_{a,b,c}^{\prime }\left(x,y,z\right) & := & \frac{1}{\left(a-c)(a-b)(1-a\right)}\left(1-x+y-z\right),\\ \chi_{a,b,c}^{\prime }\left(x,y,z\right) & := & \frac{1}{\left(b-a)(b-c)(1-b\right)}\left(1-x^{\log_{a}b}+y^{\log_{a}b}-z^{\log_{a}b}\right),\\ \psi_{a,b,c}^{\prime }\left(x,y,z\right) & := & \frac{1}{\left(c-a)(c-b)(1-c\right)}\left(1-x^{\log_{a}c}+y^{\log_{a}c}-z^{\log_{a}c}\right). \end{array}$$

In the light of Equation (43), descriptions (48) and (51) are equivalent. Analogically to the description of (48), we provide a plot of an attractor in the case of description (50).

**Figure 6:**
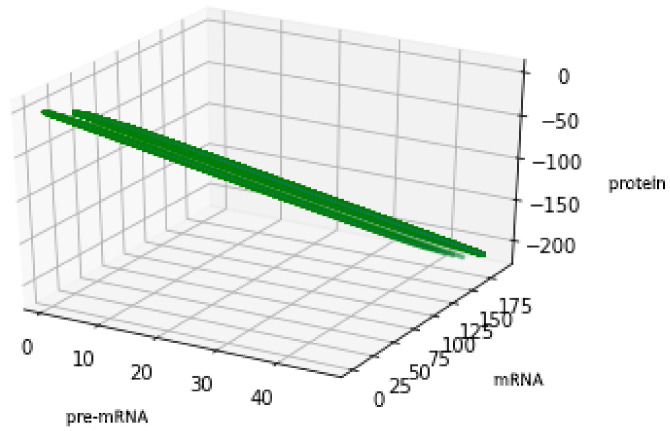
Figure presents the set described by formula (48) with the values of parameters: $a=\frac{1}{2}$, $b=\frac{3}{4}$, $c=\frac{2}{3}$. Compare with Figure 3 and Figure 5.

**Figure 7:**
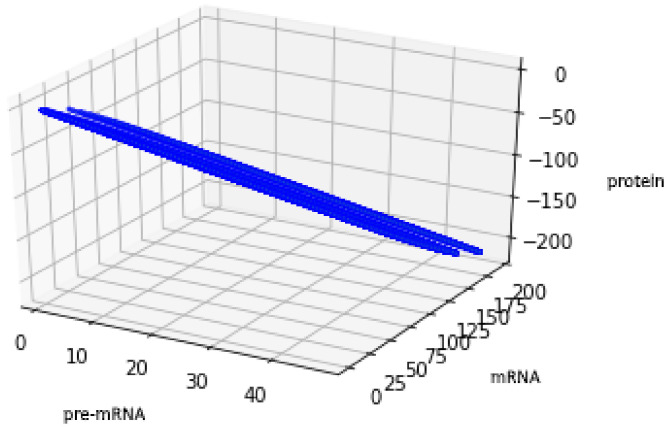
Figure presents the set described by Formula (50) with the values of parameters: $a=\frac{1}{2}$, $b=\frac{3}{4}$, $c=\frac{2}{3}$.

For the geometric reasons two Formulas (48) and (50) describe the same set A, see Figure 6 and Figure 7, compare also with Figure 5.

Now, let V={(x,y,z):1>x>y>z>0} and $f:V\to R^{3}$ be given as follows:

$$f\left(x,y,z\right)=(\varphi_{a,b,c}\left(x,y,z\right),\chi_{a,b,c}\left(x,y,z\right),\psi_{a,b,c}\left(x,y,z\right)),$$

where we use the notion taken from (49). As with the considerations in Appendix A in the paper [17] we prove that the function *f* is a local diffeomorphism. Hence $f\left(V\right)=\{x=\pi_{t_{3}}^{0}\pi_{t_{2}}^{1}\pi_{t_{1}}^{0}\left(v\right):t_{1},t_{2},t_{3}>0\}$ (see Equations (46) and (47)) is an open set. Moreover, f(V) is the interior of A. Please note that

$$\begin{array}{ccc} A_{0} & = & \{\left(\varphi_{a,b,c}\left(x,y,z\right),\chi_{a,b,c}\left(x,y,z\right),\psi_{a,b,c}\left(x,y,z\right)\right):1>x>y>z=0\},\\ A_{1} & = & \{\left(\varphi_{a,b,c}\left(x,y,z\right),\chi_{a,b,c}\left(x,y,z\right),\psi_{a,b,c}\left(x,y,z\right)\right):1=x>y>z>0\},\\ A_{0}\cap A_{1} & = & \{\varphi_{a,b,c}\left(x,y,z\right),\chi_{a,b,c}\left(x,y,z\right),\psi_{a,b,c}\left(x,y,z\right)):1>x>y=z>0\}=\\ & = & \{\varphi_{a,b,c}\left(x,y,z\right),\chi_{a,b,c}\left(x,y,z\right),\psi_{a,b,c}\left(x,y,z\right)):1=x=y>z>0\}. \end{array}$$

Hence set A is bounded by the surfaces $A_{0},A_{1}$, which are built from the trajectories of the system (36), where *i* was switched only once. The set A is indeed the support of stationary distribution when time goes to infinity. For this purpose, it is sufficient to show that:

(1) after more than two switches the trajectories of the process do not leave A,
(2) we cannot find any invariant subset B of A not equal to A. To satisfy the second condition it is sufficient to show that all the states in A communicate with each other, i.e., we can join any two arbitrary states by some trajectory of the process. The proof follows the same lines as in [17], (pp. 31–33).

## 11. Conclusions

We developed a model of gene expression using IFS with place-dependent probabilities. As a novelty, in this paper, we introduced new formulas for life-span functions, suitable for discrete case. Moreover, we have shown that asymptotic behavior of the model is in line with the results presented in the paper [17]. We have been able to perform extensive numerical simulations and describe a support of the invariant measure of this process. Both continuous-time and discrete-time system are asymptotically stable. We believe further research could find a relationship between supports of respective invariant measures. Fitting suitable values of parameters can allow use of this model along with experimental data obtained in the laboratory conditions, realistically for selected values in some time interval.

## Data Availability

Not applicable.
